# Coupling of transient near infrared photonic with magnetic nanoparticle for potential dissipation-free biomedical application in brain

**DOI:** 10.1038/srep29792

**Published:** 2016-07-28

**Authors:** Vidya Sagar, V. S. R. Atluri, A. Tomitaka, P. Shah, A. Nagasetti, S. Pilakka-Kanthikeel, N. El-Hage, A. McGoron, Y. Takemura, M. Nair

**Affiliations:** 1 Center for Personalized Nanomedicine/Institute of Neuroimmune Pharmacology, Department of Immunology, Herbert Wertheim College of Medicine, Florida International University, Miami, Florida 33199, USA; 2 Department of Biomedical engineering, College of Engineering and Computing, Florida International University, Miami, 33174 Florida, USA.; 3 Department of Electrical and Computer Engineering, Yokohama National University, Yokohama 240-8501, Japan.

## Abstract

Combined treatment strategies based on magnetic nanoparticles (MNPs) with near infrared ray (NIR) biophotonic possess tremendous potential for non-invasive therapeutic approach. Nonetheless, investigations in this direction have been limited to peripheral body region and little is known about the potential biomedical application of this approach for brain. Here we report that transient NIR exposure is dissipation-free and has no adverse effect on the viability and plasticity of major brain cells in the presence or absence superparamagnetic nanoparticles. The 808 nm NIR laser module with thermocouple was employed for functional studies upon NIR exposure to brain cells. Magnetic nanoparticles were characterized using transmission electron microscopy (TEM), X-ray diffraction (XRD), dynamic laser scattering (DLS), and vibrating sample magnetometer (VSM). Brain cells viability and plasticity were analyzed using electric cell-substrate impedance sensing system, cytotoxicity evaluation, and confocal microscopy. When efficacious non-invasive photobiomodulation and neuro-therapeutical targeting and monitoring to brain remain a formidable task, the discovery of this dissipation-free, transient NIR photonic approach for brain cells possesses remarkable potential to add new dimension.

Magnetic nanoparticles (MNPs) have been intensively investigated for various biomedical applications which includes therapeutic drugs targeting, gene delivery, bio-separation of biological entities, hyperthermia induced destruction of cells and tumors, magnetic resonance imaging (MRI), stem cell tracking, tissue repair, bio-sensing, etc.^1^,^2^,^3^,^4^,^5^,^6^,^7^,^8^,^9^,^10^,^11^,^12^,^13^. MNPs possess a distinct advantage over other nanocarriers because of their inherent superparamagnetism which allows control over its magnetization and therefore its movement/speed can be regulated. By applying remote, non-invasive magnetic forces of required intensity at the desired site it is possible to achieve tissue/cell-specific targeting with MNPs. Other characteristics of MNPs which make them popular are feasibility in production^14^ that they can be used as a contrast agent for MRI^4^,^14^, and their amphoterism in aqueous medium^15^,^16^. In aqueous solution, MNPs develop a positive or negative charge at the surface-water interface in a pH-dependent manner which allows ionic bonding of varieties of molecules at their surface^17^. Higher immobilization of molecules on MNPs can be achieved by coating or functionalization of MNPs with various surfactants^4^. Thus, the well-defined and rigid structures of MNPs serve as a solid binding platform for various ligands of diagnostic or therapeutical importance. MNPs can also be encapsulated in liposomes to create magnetoliposomes^18^. This can prevent MNPs bound drugs from direct exposure to phagocytic cells of reticuloendothelial system and other detrimental enzymatic activity in blood circulation and, in turn, physiological bioavailability of therapeutics can be significantly increased. Importantly, external control over the movement of MNPs exponentially improves the ability of the nanocarrier to reach the target site by reducing its peripheral circulation time compared to other nanocarriers^3^. Moreover, the iron content in MNPs–in particular the magnetite and maghemite- can be readily metabolized by cellular regulation using the transferrin pathway. This makes MNPs easily degradable and able to pass in and out of cells across the plasma membrane^19^. Thus, MNPs within the permissible dose limit should have non-significant safety concerns and can be extremely suitable for *in vivo* applications^20^.

In the past decade, several studies have been carried out on the development of stimuli responsive materials or techniques to design stimuli-responsive nano-devices for biomedical applications. These devices can be sensitive to a range of stimuli, which include change in pH, glutathione concentration or enzyme concentration, changes associated with the pathological situation, and extracorporeal physical stimuli via photo-, thermo- or ultrasound-targeting. These stimuli cause specific protonation, hydrolytic cleavage, molecular or supramolecular conformational changes in the material to exert the desired effect^21^,^22^,^23^. Laser-initiated photo-targeting has shown tremendous potential for cancer therapy, gene delivery, imaging, and on-demand drug delivery^24^,^25^,^26^,^27^. In most cases phototargeting is achieved by hybridizing a light source with other existing techniques. As such light sensitive hydrogels^28^,^29^,^30^ and liposomes^31^ have been discovered in recent years. Some studies used light in the UV and visible spectral range for optoporation of macromolecules in cells^32^,^33^,^34^. However, light in the UV-visible range potentiates damage to the cellular organelles, DNA and proteins. Moreover, deeper penetration of light in the UV-visible wavelength into *in vivo* tissues or organs is not possible due to higher scattering and absorption. Recently, near infrared (NIR) region light in the wavelength range of 700–1000 nm has been experimented for several biological applications. This wavelength range is referred to as transparency “therapeutic window” because of deeper *in vivo* penetration and minimum absorption and scattering in compare to UV-VIS light^35^,^36^,^37^,^38^,^39^. Nonetheless, second (1100–1350 nm) and third (1600–1870 nm) NIR spectral window may be more superior^40^. Different energy levels of NIR light beam are applied from femtoseconds to several minutes as per the necessity of application^19^,^26^,^30^,^37^,^40^,^41^,^42^.

NIR phototargeting, in conjugation with MNPs, has largely been restricted for peripheral cancer therapy by photothermal effects where targeted irradiation is applied for more than 15 minutes^19^,^42^,^43^,^44^. Considering the sophistication and interdependence of brain cells networks in driving nuances of body physiology a damaging thermal effect should be minimized or avoided while targeting brain. As such, transient or intermittent NIR exposure to brain cells can be more accommodating for their physiological ambience. A recent study suggests MNPs-NIR assisted improved gene delivery with no cytotoxicity^26^. Similarly, a magnetic/NIR-responsive on-demand, targeted drug delivery and multicolor imaging system have been invented^27^. Again, applications of this unique (combined) approach have been limited to peripheral body regions. Almost all neurological disorders remain untreated, primarily due to lack of a technique that can deliver therapeutic devices for disease diagnosis and/or treatment across the impenetrable blood-brain barrier (BBB) as and when required. Several ongoing studies showing safe use of MNPs for imaging diagnosis, drug delivery, etc. in the brain region remain at the pre-clinical stage. An improvement by combining magnetic and NIR-responsive techniques may be beneficial in this regard. The application can range from brain cell specific gene delivery, imaging and on-demand drug targeting to magnetized photobiomodulation for treating various neuro-disorders. Nonetheless, physiological implications of combined MNP/NIR phototargeting on different brain cells need to be examined. As such, we studied the effect of NIR exposure on different brain cells with or without MNP treatment. Herein, for the first time, we report that short exposure of NIR light with a wavelength of 808 nm does not affect the viability and growth behavior of three major brain cells, namely, human primary astrocytes, the SKNMC neuronal cell line, and CHME microglia cell lines. Also, combined MNP/NIR phototargeting did not affect the spinal plasticity of SKNMC neuroepithelioma cells. Thus, we believe that this combined approach can be of safe for their potential in varieties of CNS related biomedical application.

## Materials and Methods

### Synthesis of magnetic nanoparticles

The co-precipitation method was used for synthesis of magnetic nanoparticles^18^. Briefly, 3 ml FeCl_3_ (0.487 g dissolved in 2 mol l^−1^ HCl) was thoroughly mixed in 10.33 ml H_2_O and subsequent drop-by-drop addition of 2 ml Na_2_SO_3_ (0.126 g in 2 ml of water) to this solution was stir-mixed within a minute. Gradually the reaction solution turns from yellow to red-light yellow. Now 80 ml of ammonium hydroxide solution (0.80 mol^−1^) is added with vigorous stirring which lead to black precipitation. The solution is kept under continuous stirring for additional 30 minutes. The resultant MNPs crystals are washed and suspended in H_2_O which measures a pH of 7.5. The stability of MNPs can be achieved by adjusting the pH to 3.0 and subsequent heating at 90 °C and 100 °C for 5 and 60 min, respectively. All process was performed at room temperature.

### Characterization of magnetic nanoparticles

Structural conformation of synthesized MNPs was verified using Bruker GADDS/D8 X-ray diffraction system with Apex Smart CCD Detector and Mo direct-drive rotating anode (50 kV; 20 mA). Diffraction patterns were analyzed and indexed using ICDD PDF 2015 database and Match software. Further, to confirm the elemental composition of MNPs, energy dispersive spectroscopy (EDS) was conducted in scanning electron microscopy (JEOL JSM 5900LV) at 15 kV and working distance of 10 mm.

The hydrodynamic radius and size distribution of MNPs were analyzed using dynamic laser scattering (DLS) (90 Plus particle size analyzer, Brookhaven Instruments, USA) at room temperature. Further, to examine the original crystal size, transmission electron microscopy (TEM) analysis was performed with the JEOL 1010 Transmission Electron microscope operated at 100 kV. The magnetization curve of MNPs was measured using vibrating sample magnetometer (VSM-3, Toei Kogyo, Tokyo, Japan) equipped with an electromagnet (TEM-WFR7, Toei Kogyo, Tokyo, Japan) and a gaussmeter (Model 421, Lake Shore Cryotronics, Inc.). The measurement was conducted at room temperature with a maximum field of 780 kA/m.

The Agilent 8453 UV-Visible Spectrometer with Quartz-1 cm path length was used for evaluating absorbance of MNPs from 200 to 1000 nm wavelength.

### Cell culture

SK-N-MCs, a neuroepithelioma cell line derived from a metastatic supra-orbital human brain tumor, were cultured in minimum essential medium (MEM). MEM was supplemented with 10% fetal bovine serum (FBS), 100 U/ml penicillin, and 100 mg/ml streptomycin (Gibco-BRL, Gaithersburg, MD). Cells were incubated at 37 °C in a 5%CO_2_ incubator. Similarly, human primary astrocytes (HA) and CHME-5 human microglia cells were cultivated as per provider’s recommendations.

### NIR Exposure

NIR exposure was performed with collimated NIR Laser Module source (RLDH808–1200-5, Roithner Laserthchnik Gmbh, Vienna, Austria) as described by Tang and McGoron^45^. 808 nm NIR with ~1.5 W/cm^2^ power density was focused for 2 minutes on brain cells (human primary astrocytes, SKNMC neuronal cells, and CHME-5 glia cells) cultured in 96 well plates in the presence or absence of MNPs (50 μg MNPs/ml). This fixed laser source has spot size of 5 mm which approximately covers central 80% cells of a well in 96 well culture plates. Cells in the periphery of a well are exposed due to potential beam-spread upon surface hitting of NIR during irradiation and as such whole well is illuminated (Supplementary Fig. 3). Cells were cultured in alternate wells so that potential cross-talk of NIR to a specific well was minimized for an adjacent well. Cells were pre-treated with MNPs 12 hrs before NIR targeting. Temperature of a specific well was measured using a thermocouple (0.22 mm diameter) for the entire 0–2 min of NIR exposure.

### Cell viability assay

The MTT (Thiazolyl blue tetrazolium bromide) cell proliferation assay was performed as described previously^17^,^18^,^46^. Briefly, cells after NIR treatment were re-incubated at 37 °C for 3–6 hours (to imitate a real-time situation where cells will be under the natural condition post NIR treatment). Cells from different experimental groups were given a 200 μl media change with 20 μl MTT solution added and gently rocked in the dark at room temperature for 2–3 hrs. One volume of STOP solution containing 20% SDS in 50% dimethyl formamide was added to the rocking cell suspension in MTT solution and further gently rocked in the dark at room temperature for 1–2 hrs. The cell suspension was centrifuged at 2000 rpm for 10 minutes and the supernatant was collected for the optical density determination of the solubilized formazan at 550 nm using Spectronic Genesys Bio10 spectrophotometer. The optical density of formazan in each treatment groups is directly proportional to the cell viability.

### Cell growth resistance/impedance (Ω) measurement

Astrocytes growth resistance/impedance was measured with the help of the Electric cell-substrate impedance sensing instrument (model 1600RE, Applied Biophysics, USA) using 8W10E PET chips (Applied Biophysics), containing 8 cell culture wells^47^,^48^. Each well of the chip contains 10 working electrodes (250 μm diameter) embedded in parallel on a gold connection pad and all wells share a common reference electrode. Astrocytes cultured with or without MNPs treatment were photo-targeted with NIR light and seeded in chip wells (5 × 10^4^ cells/well). Both, the working and the reference electrodes were connected to a phase-sensitive lock-in amplifier through a 1 MΩ resistor before applying the AC signal. An electric potential of 1 V at 4 KHz was used for cell growth resistance measurements at 37 °C in a humidified incubator for 0–10 hrs.

### Confocal microscopy and Characterization of neuro-spine density

Membrane staining of neuronal cells for confocal microscopy and measurement of spine density was performed according to the method adopted from Atluri *et al*.^49^. Cells were imaged using TCS SP2 Confocal Laser Scanning Microscope (Leica Microsystems, Germany) at 488 nm using 60X oil immersion objectives and 2.5X confocal electronic zoom.

### Biostatistical analysis

Data in different figures are presented as mean ± standard error of three experiments (n = 3). Student’s t-test was performed to compare means of two groups using GraphPad prism6 (San Diego, Ca) and *P* values ≤0.05 were considered as significant.

## Results and Discussion

### Short-term MNPs-NIR exposure does not affect the temperature of cell culture ambience

**Figure 1:**
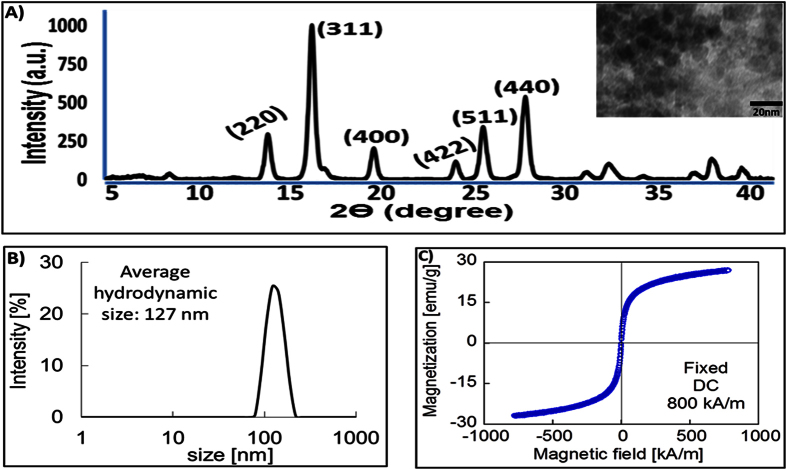
Characterization of magnetite nanoparticles. (**A**) XRD spectrum of <20 nm MNPs (in TEM insert image at top right hand side) showing magnetite-specific characteristics plane. (**B**) Dynamic laser scattering (DLS) measurement of hydrodynamic size distribution of MNPs shows an average colloidal size of nanoparticles is 127 nm. (**C**) Magnetic hysteresis loop of MNPs showing superparamagnetism i.e. zero coercivity at room temperature.

We herein investigated the combined effect of MNPs and NIR treatment on growth dynamics of three major brain cells *i.e.* astrocytes, microglia and neuronal cells. MNPs were synthesized using the co-precipitation method which is regarded as one of the most efficient ways to prepare MNPs. In the co-precipitation method, either Na_2_SO_3_ or FeSO_4_ is used to reduce ferrous ion from FeCl_3_. While FeSO_4_ based reduction results in rod-shaped nanoparticles, the relatively gentle reduction ability of Na_2_SO_3_ in an aqueous solution produces round MNPs. The primary product of this reduction reaction is magnetite, which can be further acid-oxidized at 100 °C for maghemite as the more chemically stable end product. Nonetheless, both magnetite and maghemite has similar magnetic properties^50^. The crystalline structure and phase purity of synthesized nanoparticles were evaluated using x-ray diffraction spectroscopy which shows magnetite/maghemite specific diffraction peaks (220, 311, 400, 511, and 440 planes; JCPDS 00-089-0691) (Fig. 1A). Further, Energy Dispersive X-Ray Spectorscopy (EDS) analysis confirmed FeO specific elemental composition. Observation of both FeL and FeK peaks for Fe_3_O_4_ in EDS is an expected outcome because many elements can be observed with more than one shell in a specific energy range (Supplementary Fig. 1). The polydispersity index of 0.19 in DLS suggests a very narrow size distribution of these particles. Nonetheless, average hydrodynamic size was estimated as 127 nm (Fig. 1B) which is higher than the TEM size of <15 nm (Insert image in Fig. 1A). This size difference between TEM and DLS can be attributed to dried and aqueous solution of particles used during respective analysis. The hydrodynamic size of nanoparticles in colloidal suspension is always greater than TEM due to adsorbed aqueous molecules. Water molecules (H-O-H) influence the surface charge (Fe-OH + H^+^ = Fe-OH_2_^+^/Fe-OH = Fe-O^−^ + H^+^) which allows binding of molecules (e.g. drugs, nucleic acid, etc.) to MNPs via ionic interaction for various biomedical applications. The magnetic hysteresis loops of these particles display high saturation magnetism at room temperature when measured between +780 and −780 Oe (Oersted). The magnetization curves displayed typical superparamagnetic behavior with no hysteresis and zero coercivity (Fig. 1C). The superparamagnetism can be utilized to manipulate MNPs movement for target-specific delivery by an external, non-invasive magnetic force and MNPs of this size possess several advantages for neurotargeting. Such as, it is possible to manipulate and target at the subcellular organelles levels^51^. Their higher surface to volume ratio increases loading efficiency of therapeutical agents on MNPs surfaces. More importantly, MNPs between 10–70 nm can readily penetrate capillary vessels^17^ and therefore it serves as a compatible therapeutical carrier that can transmigrate across the tightly junctioned brain microvascular endothelial cells (BMECs) of BBB along the capillary lining throughout the cerebral microvasculature. Even with coatings of NIR responsive materials, such as hydrogels, liposomes, etc. on MNPs, the BBB transmigration ability of the nanoformulations is expected to be maintained. Magnetoliposomes up to 150 nm size have been reported to cross *in vitro* BBB by the application of 0.3 Tesla magnetic fields^52^. It is expected that the application of a magnetic field dissipate energy into magnetic particles resulting in a thermal impact on the targeted area. Nonetheless, the heating power of MNPs depends on the intensity and frequency of alternating magnetic field and MNPs size and geometry^53^,^54^,^55^. Particles within 30 nm size exert zero to minimum hyperthermia due to the magnetic field^48^. Also, many investigations have shown that MNP transmigration across the BBB using an external magnetic force has no adverse effect on the integrity of tight junctions^9^,^10^,^11^,^12^,^13^,^17^,^18^,^46^,^56^,^57^.

**Figure 2:**
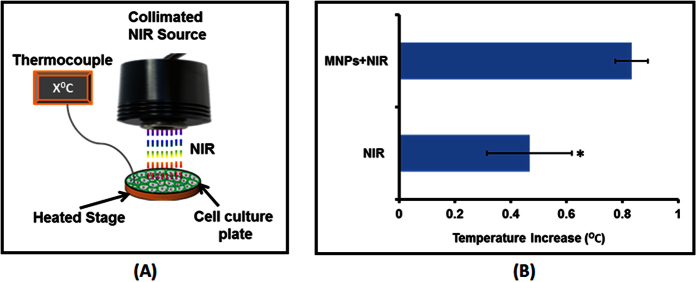
Temperature profiling during NIR exposure. (**A**) Schematic of 808 nm NIR laser module: NIR source is fixed to a holder and connected to an external power supply. Beneath the NIR source is a heated stage insert to keep the cell-culture system at original culture of 37 °C. Temperature of cell culture system exposed to NIR with or without MNPs is measured with the help of a thermocouple wire. (**B**) Temperature profile of different experimental group: Cell culture with or without MNPs were exposed with ~1.5 Wcm^−2^ NIR for 2 minutes and temperature were recorded via placing thermocouple at the bottom of well throughout the exposure period. Effect of NIR or MNPs-NIR on temperature of cell culture ambience of individual well was obtained by temperature difference at the beginning (0 second) and end (120 seconds) of exposure. NIR exposure on cell culture system without MNPs showed a temperature rise of 0.46 ± 0.152 °C and that of with MNPs showed an increase of 0.83 ± 0.057 °C (*P < 0.0177).

NIR-based irradiation in the presence of MNPs was achieved using the collimated NIR laser delivery system as shown in Fig. 2A. The system has been previously used to investigate the *in vitro* effect of the combined therapeutic modalities of chemotherapy and hyperthermia to cancer cell lines^45^. The NIR spectrum in the range of 800–1000 nm wavelength, itself, does not induce DNA damage and is non-toxic to tissues. However, heat may be released following exposure when a material that absorbs energy in the NIR region is present^58^,^59^. As such, NIR phototargeting in the presence of MNPs may lead to a photothermal effect which primarily depends on the duration and dose of NIR exposure and nanoparticle cluster density^26^,^44^,^42^,^53^. Studies suggest that *in vivo* cytotoxic effects due to hyperthermia are exerted only when NIR with higher power (>3 Wm^−2^) is exposed for several minutes on a daily basis for several weeks in the presence of MNPs^53^. In fact, a study by Chu *et al*.^42^ shows that NIR exposure for less than 3 minutes on MNP treated cells does not induce any damage or adverse effect^42^. Short-term NIR exposure in conjugation with MNPs can be successfully applied for the gene delivery, imaging, and controlled drug delivery^26^,^27^. As a first step towards assessing the efficacy of this approach for the brain region, we evaluated the effect on temperature change of an *in vitro* cell culture system upon short-term NIR light exposure (up to 2 minutes). The 808 nm NIR laser has been demonstrated to have superior CNS tissue penetration compared to light of other wavelengths such as 606 nm and 940 nm^60^. As such we selected this wavelength for our experiments. In clinical NIR based transcranial photobiomodulation ≤2 minute exposure of 808 nm NIR exposure has been shown efficacious in treating major depressive disorder^61^,^62^. Schematic in Fig. 2A shows the cell culture plate in the presence or absence of MNPs, which were homogenously exposed to 808 nm NIR with power ~1.5 Wcm^−2^. Typical power intensities reported for photothermal treatment range from 1 to about 100 Wcm^−2^ 63. Since no previous reports suggest combine use of MNPs and NIR for brain cells, a minimum recommended power density was selected considering higher physiological sensitivity of brain cells in compare to peripheral cells. To simulate the original cell culture condition, the temperature was maintained at 37 °C using the heated platform upon which the culture plates were placed during the NIR laser exposure and increase or decrease in temperature was measured using a thermocouple. As shown in Fig. 2B, NIR laser exposure on the cell culture system without MNPs showed a temperature rise of 0.46 ± 0.152 °C and that with MNPs showed an increase of 0.83 ± 0.057 °C. MNPs absorption at 808 nm is near to bottom line i.e. extremely low (Supplementary Fig. 3). Nonetheless, laser beam energy absorbed by the components of cell-culture media may also add to the 0.46 °C temperature rise in the absence of MNPs. Thus, the net temperature rise upon NIR laser exposure in the presence of MNPs is only 0.37 ± 0.115 °C which is insignificant in terms of hyperthermia generation and can be fairly safe to use in an *in vivo* situation.

### Short-term MNPs-NIR exposure does not affect the brain cell viability, growth behavior and plasticity

**Figure 3:**
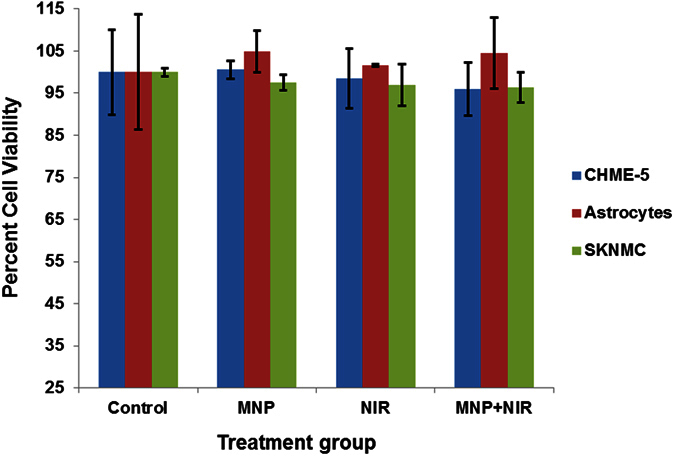
Percent cell viability of three major brain cells, namely, Human primary astrocytes, SKNMC human *neuroepithelioma* cells, and CHME-5 human microglia cell lines were obtained using MTT cytotoxicity assay. None of the treatment affected the viability of either of cell lines. Percent cell viability in the case of NIR-alone experiment was 98.45 ± 7.05, 101.58 ± 0.36, and 96.90 ± 4.85 for CHME-5 human microglia cell lines, Human primary astrocytes and SKNMC human *neuroepithelioma* cells and that for MNPs-NIR treatment was 96.00 ± 6.25, 104.58 ± 8.40, and 96.35 ± 3.64.

In order to assess the potential use for the brain region, we examined the nonspecific cytotoxicity of this novel MNPs-NIR during short-time (808 nm for 2 minute) treatment to three major brain cells, namely, Human primary astrocytes, SKNMC human neuroepithelioma cells, and CHME-5 human microglia cells. The results of quantitative cell cytotoxicity determined by the MTT cell proliferation assay showed that MNPs-NIR exposure was not toxic to any of the brain cell types used in this study (Fig. 3). Similar to our previous report^17^, MNPs alone did not deter the cell viability of Human primary astrocytes, SKNMC human *neuroepithelioma* cells or CHME-5 human microglia cells. NIR light with 800–100 nm wavelength can be transmitted across tissue without inducing any damage to cellular macromolecules or organelles. As such, similar to MNPs-alone treatment, cell viability is expected to remains unaltered upon exposure of NIR-alone. Percent cell viability in the case of NIR-alone experiment was 98.45 ± 7.05, 101.58 ± 0.36, and 96.90 ± 4.85 for CHME-5 human microglia cells, Human primary astrocytes and SKNMC human *neuroepithelioma* cells, respectively. Similar to this result, it has been shown previously that 808 nm irradiation on Eca-109 cells for 20 minutes did not change their viability^48^. Shen *et al*.^19^ also showed the same for the A549 cells which were exposed to a 5 Wcm^−2^ 808 nm NIR laser in the presence or absence of individually dispersed MNPs for 3 minutes^19^. As shown in Fig. 3, percent cell viability in the case of MNP-NIR treatment was 96.00 ± 6.25, 104.58 ± 8.40, and 96.35 ± 3.64 for CHME-5 human microglia cells, Human primary astrocytes and SKNMC human neuroepithelioma cells, respectively. The unaffected cell viability either during NIR-alone or MNPs-NIR exposure (Fig. 3) coincides with the insignificant temperature rise of the culture system (Fig. 2b). In fact, studies pertaining to the oncolytic effect of MNP-NIR exposure report that the cytotoxic effect is induced when the local temperature rise is greater than 42 °C 4,28. The “no-effect” exhibition of cell viability in this study may also be because of the low light absorption by the natural endogenous cytochromes of cells upon short-term exposure, which may cause minimal temperature elevation accounting to the high cell survivability^19^. The unaffected percent cell viability potentiates the safe use of short-term MNP-NIR exposure for biomedical applications in the brain region.

**Figure 4:**
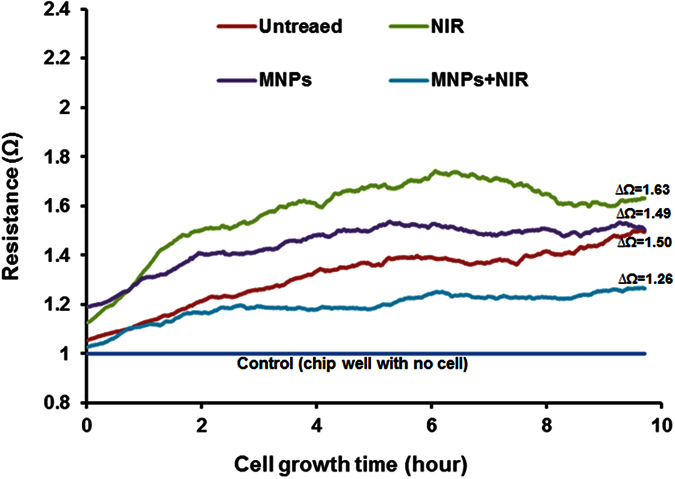
Cell growth behavior measurements for human primary astrocytes over 10 hours of culture: Growth-resistance (Ω) kinetics of astrocytes with all kinds of treatments showed a steady upward slope. The end point blank-normalized resistance (∆Ω) values were 1.63, 1.49, 1.26, and 1.50 for NIR-alone treatment, MNPs-alone treatment, MNPs-NIR, and untreated control exposure, respectively. Thus, the cell growth-resistances patterns were in the same range which suggests a similar growth behavior of attached cells across all groups.

While the MTT cell proliferation assay provides a general sense of cytotoxicity at a final time point after specific duration of treatment, analyzing the continuous growth behavior of cells over time may reveal kinetic effects of toxicity^23^,^24^. As such, astrocytes growth resistance/impedance was measured for 10 hours post-treatment using the electric cell-substrate impedance sensing method. This method measures the resistance (Ω) produced by growing cell monolayers over the electrodes and can detect changes in resistance to AC current flow that may occur with changes in the cell layer^47^,^48^. Primarily, growth resistance of each treatment group is compared with resistance of blank well containing culture media to obtain blank-normalized resistance value. As shown in Fig. 4, growth-resistance kinetics of astrocytes with all kinds of treatments showed a steady upward slope. The end point blank-normalized resistance (ΔΩ) value after 10 hrs of culture was 1.63, 1.49, 1.26, and 1.50 for NIR-alone treatment, MNPs-alone treatment,MNPs-NIR and untreated control exposure, respectively. Thus, the cell growth-resistances values were in the same range, which suggests a similar growth behavior of attached cells across all groups. The presence of MNPs on the electrode surface of the culture chip may result in a slower cellular attachment process; however, in a real in vivo scenario the effect on the cellular attachment process due to MNPs may be inconsequential. Moreover, the intrinsic AC properties of MNPs may also interfere with the AC current flow of the culture chip electrodes and subsequently may obstruct the cell growth-resistance behavior via the resistance measurement.These factors may have resulted in a relatively slower kinetic slope in MNPs-only and MNPs-NIR treated cells than the NIR-only and untreated control groups. A little up or down slope of the growth kinetics among groups is expected during the early hour due to variations in the cell attachment rate which can be influenced by many other factors such as initial cell density, cellular transitory metabolic slowdown due to treatment effects, etc.^47^,^48^. Thus, a longer monitoring of cell growth-behavent, impedance sensing method may reflect a healthy cellular status and factors such as cell attachment and metabolic pause can be nullified. Nonetheless, the astrocytes growth-resistance pattern obtained for the initial 10 hrs is in accordance with the MTT assay (Fig. 3) suggesting no harmful effect of short-term MNPs-NIR exposure on cell health.

**Figure 5:**
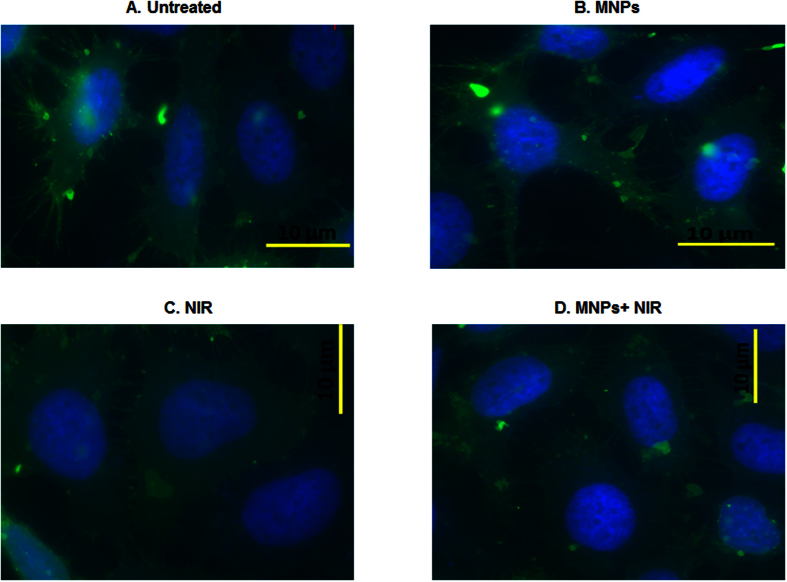
Confocal microscopy to evaluate the dendrite and spine morphology (synaptic plasticity) of SKNMC cells after 72 hours of NIR treatment in the absence (**C**) or presence (**D**) of MNPs in compare to control (**A**,**B**): Healthy dendritic and spine morphology of SKNMC cells is evident for all treatments. This suggests NIR phototargeting did not alter the neuronal synaptic plasticity and thus, similar to short-term effect (Figs 3 and 4), long term effect of MNPs-NIR phototargeting potentiate towards the safe use of this novel approach.

**Figure 6:**
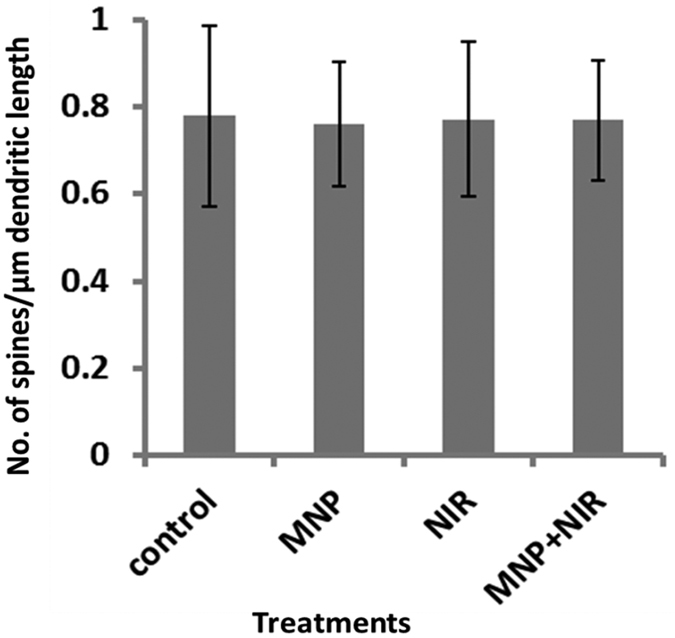
Spinal density (No. of spines/μm dendritic length) of SK-N-MC: spine density remains unchanged in all treatments (Untreated Control: 0.77 ± 0.20 per μm^2^, MNPs: of 0.76 ± 0.14 per μm^2^, NIR: 0.76 ± 0.13 per μm^2^, and MNP + NIR: 0.77 ± 0.17 per μm^2^) which show coupling of transient near infrared photonic with magnetic nanoparticle have no adverse effect on brain cell growth and behaviors.

Long term effect of MNPs-NIR treatment on dendrite and spine morphology (synaptic plasticity) of SKNMC cells was monitored. Spine morphology plays an important role in maximizing the effectiveness of the synaptic transmission in brain and to the periphery^17^,^49^. Treated or untreated cells were allowed to grow on cover slip for more than 72 hours such that homogenous elongation of dendritic and spinal projections can take place. Subsequently, cells were PBS-fixed and stained using the green-fluorescent membrane tracer 1, 1′-Dioctadecyl-3, 3,3′,3′-tetramethylindocarbocyanine perchlorate. This lipophilic dye uniformly labels lipid contents of plasma membrane via lateral diffusion. The stained cells on slides were now microscoped for confocal imaging. The 60X immersion objectives lens at 488 nm illusion and 2.5x electronic zoom could give us the required magnification to visualize dendritic spine of individual cells. Spine density were quantified using obtained confocal images using a well-established protocol^17^,^46^,^49^ where ImageJ software is used to measure defined length of single cells and no. of spines present within that length is counted (spine density = number of spines/dendritic or cell length). As shown from confocal microscopy in Figs 5 and 6, healthy dendritic and spine morphology of SKNMC cells is evident for all treatments. NIR treated cells with or without MNPs showed a spinal density of 0.76 ± 0.13 and 0.77 ± 0.17 per μm^2^, respectively, whereas the same in untreated cells were approximately 0.77 ± 0.20 per μm^2^ (Fig. 6). Cells treated with MNPs only showed a spinal density of 0.76 ± 0.14 per μm^2^(Fig. 6). We have shown earlier, similar to this case (Fig. 5B), that MNPs alone did not affect the dendritic and spinal elongation^17^,^46^. Similarly, NIR-alone (Fig. 5C) and MNPs-NIR (Fig. 5D) treated cells showed dendritic and spinal morphology comparable to that of untreated controls (Fig. 5A). Thus, healthy synaptic plasticity was observed in all experimental groups. This result further substantiates the above observations that short-term MNPs-NIR exposures do not affect brain cell growth and behaviors and, as such, this novel, combined approach may be used for biomedical applications in brain regions.

### Prospective

**Figure 7:**
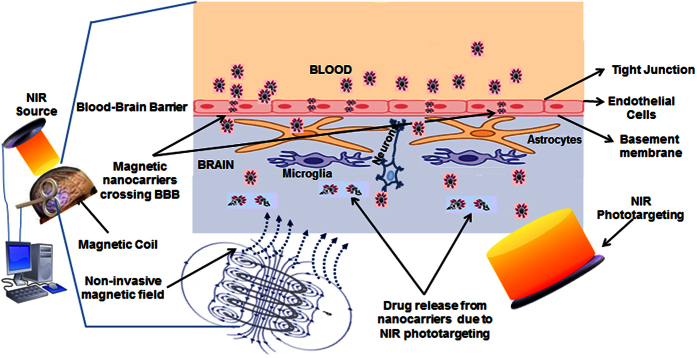
Proposed mechanism for efficacious targeting, delivery, release, and monitoring of therapeutics to brain using transient NIR photonics with MNPs for noninvasive brain targeting: An *in silico*-controlled, non-invasive magnetic force will drive MNPs across BBB and simultaneously NIR phototargeting can cause release of associated drugs (transitory optoelectronic excitation-mediated molecular vibrations on MNPs surfaces, even for short NIR laser exposure, change the charge distribution of MNPs surfaces and subsequently the ionic interaction of drugs can be broken with subsequent drug release). Also, while MNPs alone are used for MRI imaging; a “MNP-NIR sensitive fluorescent materials::core-shell” template can be an excellent use for multi-color imaging modalities. Combining fluorescent liposomes, hydrogels, carbon dots, or matrix of upconversion materials such as Erbium, Thulium, etc., can be used for this purpose because these materials can emit different color fluorescent lights depending on NIR wavelength.

We demonstrated that transient NIR irradiation in presence of MNPs is dissipation free and safe for brain cells. Our results suggest that MNPs-NIR phototargeting does not have adverse effect on the viability, growth behavior and plasticity of brain cells. Thus, selected power density of ~1.5 W/cm^2^ and 2 minute time window for 808 nm NIR exposure in this report will open a referral point to explore higher power density laser for brain cells. This opens up a regime that can provide an exceptional opportunity for the safe use of this novel combinatorial approach for various biomedical innovations. Such as, transitory optoelectronic excitation-mediated molecular vibrations on MNPs surfaces, even for short NIR laser exposure, may change the charge distribution of MNPs surfaces and subsequently the ionic interaction of drugs can be broken. Thus controlled, on-demand drug release may be achieved. Discovery of NIR-sensitive materials such as hydrogels and liposomes may further advance the applicability of this approach where drugs bound MNPs can be encapsulated in liposomes or hydrogel layers. Brain targeting requires a faster reach of drug carriers with no or little exposure to RES for maximum bioavailability. External control over MNPs movement can be highly applicable for this purpose. Encapsulation can prevent drugs from digestion by direct reticuloendothelial system (RES) exposure and enzymztic activity of the peripheral circulation (blood), can remarkably improve the total drug loading capacity of the carrier by loading drugs on MNPs and encapsulating materials as well, and can enhance the drugs stability and prevent leaching in the blood circulation. Also, drug dissemination in healthy tissues can be prevented with maximized target bioavailability. A recent study by Tedford *et al*.^60^ showed that transcranial and intraparenchymal exposure of 808 nm NIR laser can successfully penetrate 40 mm deep into the scalp, human skull and meninges. As such, short-term MNPs-NIR (with or without coating of NIR sensitive materials) phototargeting may be a new innovation for controlled, on-demand drug delivery in brain (Fig. 7) and for photobiomodulation to treat different psychological disorders. Moreover, while MNPs alone are used for MRI imaging; a “MNP-NIR sensitive fluorescent materials::core-shell” template can be an excellent use for multi-color imaging modalities. The NIR sensitive fluorescent materials may be liposomes, hydrogels, fluorescent carbon dots, or matrix of upconversion materials such as Erbium, Thulium, etc., which can emit different color fluorescent lights depending on NIR wavelength^64^. Nonetheless, sufficient research is required to establish permissible limits of different MNPs-NIR combination that have non-significant safety concerns before practical application on a real scenario.

## Additional Information

**How to cite this article**: Sagar, V. *et al*. Coupling of transient near infrared photonic with magnetic nanoparticle for potential dissipation-free biomedical application in brain. *Sci. Rep.* **6**, 29792; doi: 10.1038/srep29792 (2016).

## Supplementary Material

Supplementary Information
