# Supplementary material for: Coupling of transient near infrared photonic with magnetic nanoparticle for potential dissipation-free biomedical application in brain
Source: Sci Rep. 2016 Jul 28;6:29792. doi: 10.1038/srep29792 (PMC4964614; doi:10.1038/srep29792)

**Coupling of transient near infrared photonic with magnetic nanoparticle for potential dissipation-free biomedical application in brain**

Vidya Sagar^1, 2^, V.S.R. Atluri^2^, A. Kami^1, 2^, P. Shah^3^, A. Nagasetti^3^, S. Pilakka-Kanthikeel^1, 2^, N. El-Hage^2^, A. McGoron^3^ & M. Nair^1, 2,^ *

Affiliations:

^1^Center for Personalized Nanomedicine/^2^Institute of Neuroimmune Pharmacology, Department of Immunology, Herbert Wertheim College of Medicine, Florida International University, Miami, Florida 33199.

^3^Department of Biomedical engineering, College of Engineering and Computing, Florida International University, Miami, Florida 33174

*Correspondence to: Dr. Madhavan Nair, [nair@fiu.edu](mailto:nair@fiu.edu); and Dr. Vidya Sagar, vsaga001@fiu.edu

**Legends for supplementary figure**

Fig S-1: Energy Dispersive X-Ray Spectorscopy (EDS) analysis to confirm FeO-specific elemental composition.

Fig S-2: UV-Visible Spectra to understand absorbance of MNPs from at NIR wavelength.

Fig S-3: Picture showing illumination of whole well upon NIR exposure.

Fig S-1


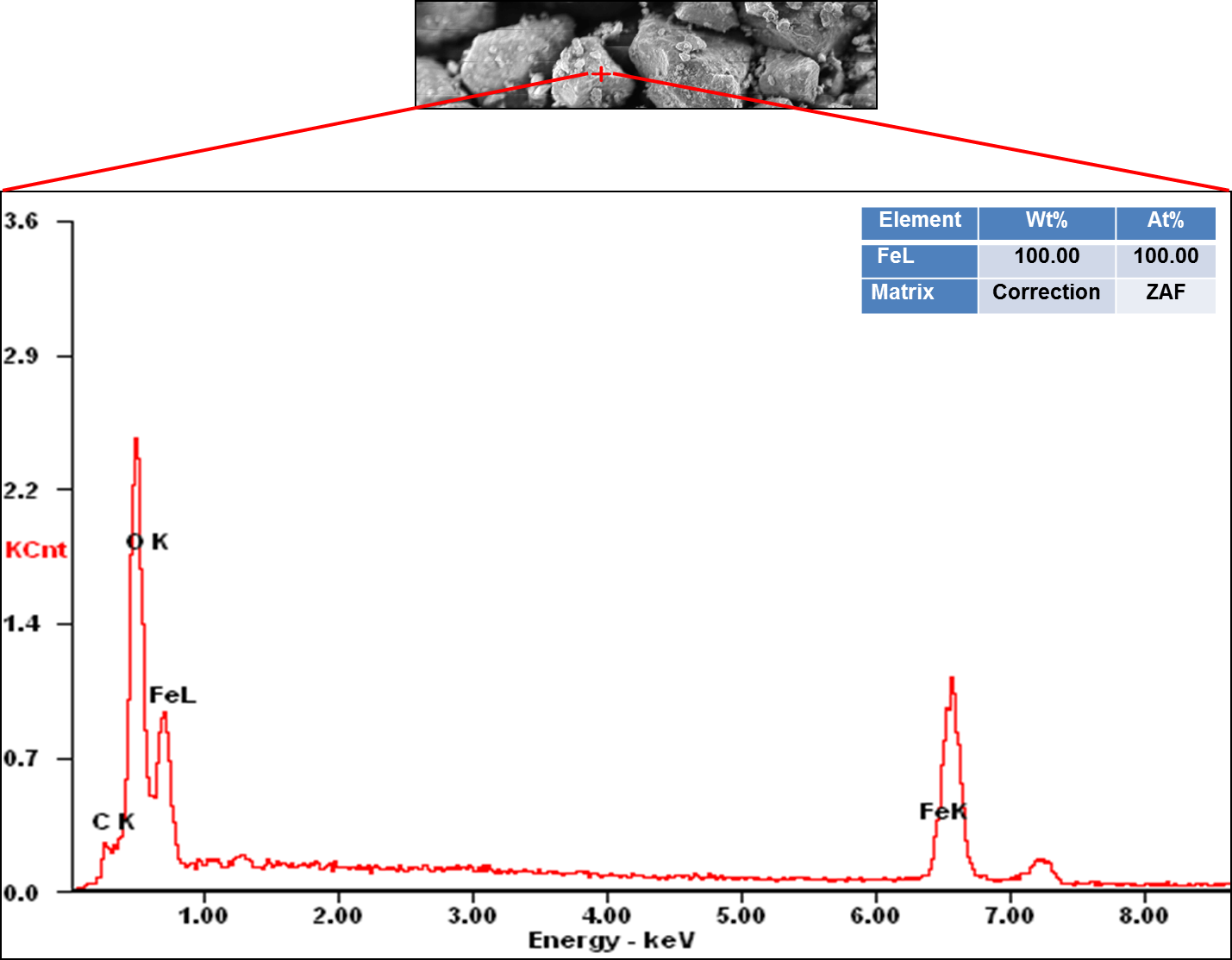


Fig S-2


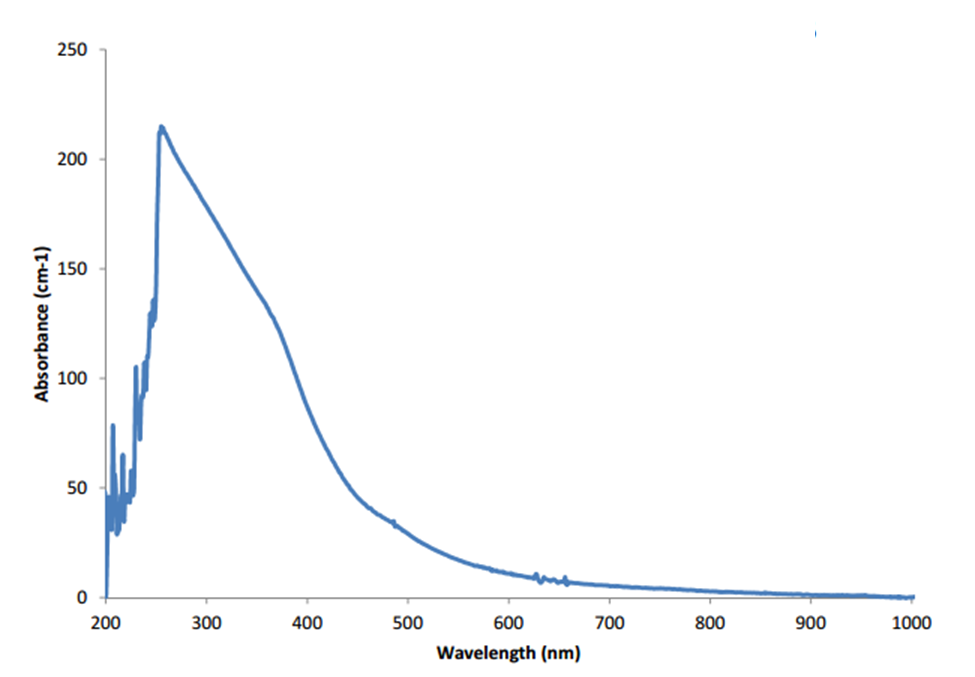


Fig S-3


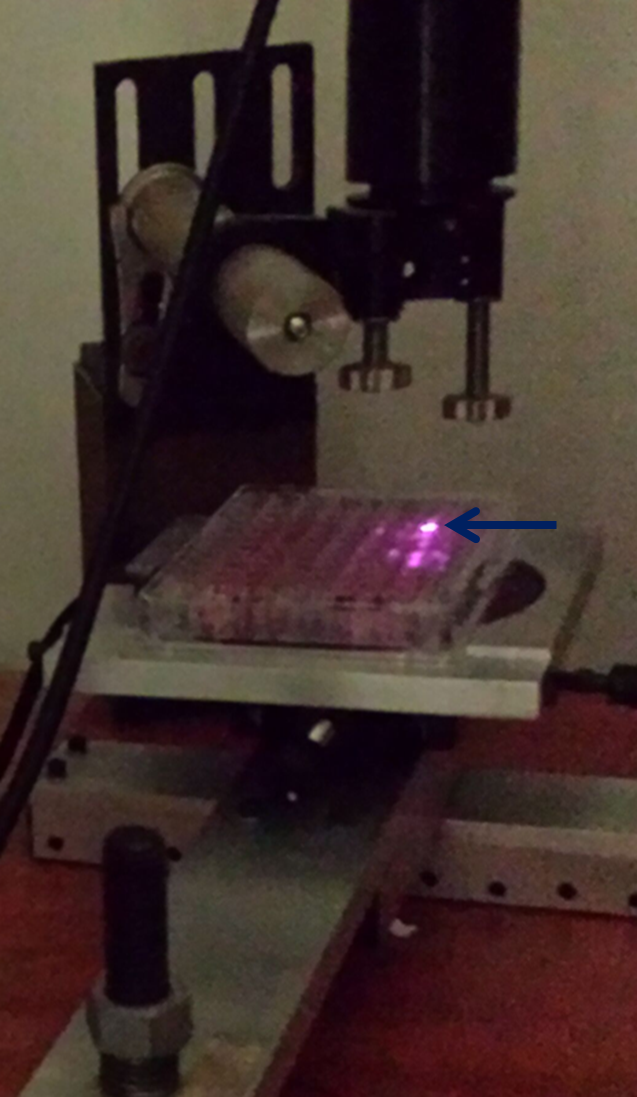

Supplement: Supplementary Information [file srep29792-s1.docx]
